# Intravital imaging of glioma border morphology reveals distinctive cellular dynamics and contribution to tumor cell invasion

**DOI:** 10.1038/s41598-019-38625-4

**Published:** 2019-02-14

**Authors:** Maria Alieva, Verena Leidgens, Markus J. Riemenschneider, Christoph A. Klein, Peter Hau, Jacco van Rheenen

**Affiliations:** 1 Hubrecht Institute-KNAW & University Medical Center Utrecht, Uppsalalaan 8, 3584 CT Utrecht, The Netherlands; 2 Prinses Máxima Center for Pediatric Oncology, Uppsalalaan 8, 3584CT Utrecht, The Netherlands; 3 Department of Neurology and Wilhelm Sander-NeuroOncology Unit, University Hospital Regensburg, Regensburg, Germany; 4 Department of Neuropathology, Regensburg University Hospital, Regensburg, Germany; 5 Department of Experimental Medicine, University of Regensburg, Regensburg, Germany; 6 Department of Molecular Pathology, Oncode Institute, Netherlands Cancer Institute, Plesmanlaan 121, 1066CX Amsterdam, The Netherlands

## Abstract

The pathogenesis of glioblastoma (GBM) is characterized by highly invasive behavior allowing dissemination and progression. A conclusive image of the invasive process is not available. The aim of this work was to study invasion dynamics in GBM using an innovative *in vivo* imaging approach. Primary brain tumor initiating cell lines from IDH-wild type GBM stably expressing H2B-Dendra2 were implanted orthotopically in the brains of SCID mice. Using high-resolution time-lapse intravital imaging, tumor cell migration in the tumor core, border and invasive front was recorded. Tumor cell dynamics at different border configurations were analyzed and multivariate linear modelling of tumor cell spreading was performed. We found tumor border configurations, recapitulating human tumor border morphologies. Not only tumor borders but also the tumor core was composed of highly dynamic cells, with no clear correlation to the ability to spread into the brain. Two types of border configurations contributed to tumor cell spreading through distinct invasion patterns: an *invasive margin* that executes slow but directed invasion, and a *diffuse infiltration* margin with fast but less directed movement. By providing a more detailed view on glioma invasion patterns, our study may improve accuracy of prognosis and serve as a basis for personalized therapeutic approaches.

## Introduction

Glioblastoma (GBM) is one of the most aggressive primary brain tumors, with a median survival time of about 14.6 months despite maximal therapy^1^. Besides resection and radiotherapy, Temozolomide, a cytotoxic drug^2^ and Optune, so-called Tumor Treating Fields^3,4^, remain the only measures that improve outcome.

GBM is hallmarked by a high complexity and heterogeneity^5,6^, making a deep understanding of its pathogenesis challenging. The tumor is driven by a minority of cancer stem-like brain tumor initiating cells (BTIC)^7,8^, that appear to be not only implicated in tumor initiation, but also in recurrence, progression^9,10^ and resistance to current therapy^8,11^. BTICs and non-stem tumor cell co-exists *in vivo* and are likely to change dynamically depending of the tumor microenvironment^12,13^. In view of modelling the disease, BTICs are the best available cell population to investigate GBM *in vitro* and *in vivo*^14,15^.

The pathogenesis of GBM is manifold and includes highly-invasive behavior that is a main cause behind GBM dissemination and progression^16,17^. There is general agreement that invasion is an early event in GBM progression^16^, where the tumor cells tend to invade individually or in small groups^17,18^. These infiltrating tumor cells lie well beyond the definable margin for maximal resections^17,18^ giving rise to tumor relapse^19^. GBM cells are thought to preferentially migrate along existing brain structures^12,18^, however vascular co-option and migration along white matter tracts are also important features of glioma cell invasion^16,20,21^. Tumor borders are not always uniform, and several invasion patterns such as single cell invasion^18,22^ and collective migration with leading and follower cells^23,24^ have been described. However it is unclear how these patterns correlate to clinically relevant macroscopic tumor growth and dissemination^18,25^.

The reason why a conclusive image of the invasive process in malignant gliomas is still not available is partly due to the lack of relevant models. Screens based on partitioned resections from tumor boarder and core^26,27^ and conventional *in vitro* migration assays^28–30^ are highly artificial and cannot recapitulate *in vivo* tumor cell behavior.

The development of intravital microcopy (IVM), a potent tool that allows to perform single-cell resolution time-lapse imaging on live animals, has provided new insights into (GBM) tumor cell dynamics^22,31–39^. To further investigate the physiological processes^40^ underlying GBM cell movement, this study aimed to image and analyze distinct GBM invasive growth patterns found *in vivo*, similar to those observed in patients. We combined an orthotopic human BTIC-derived GBM model with real time dynamic high-resolution IVM^22,31,35^ followed by a comprehensive analysis of tumor cell migratory behavior and found that distinct types of tumor border morphologies present different invasive growth patterns that can contribute to tumor expansive growth.

## Results

### Distinct tumor border configurations in glioma tumors

**Figure 1:**
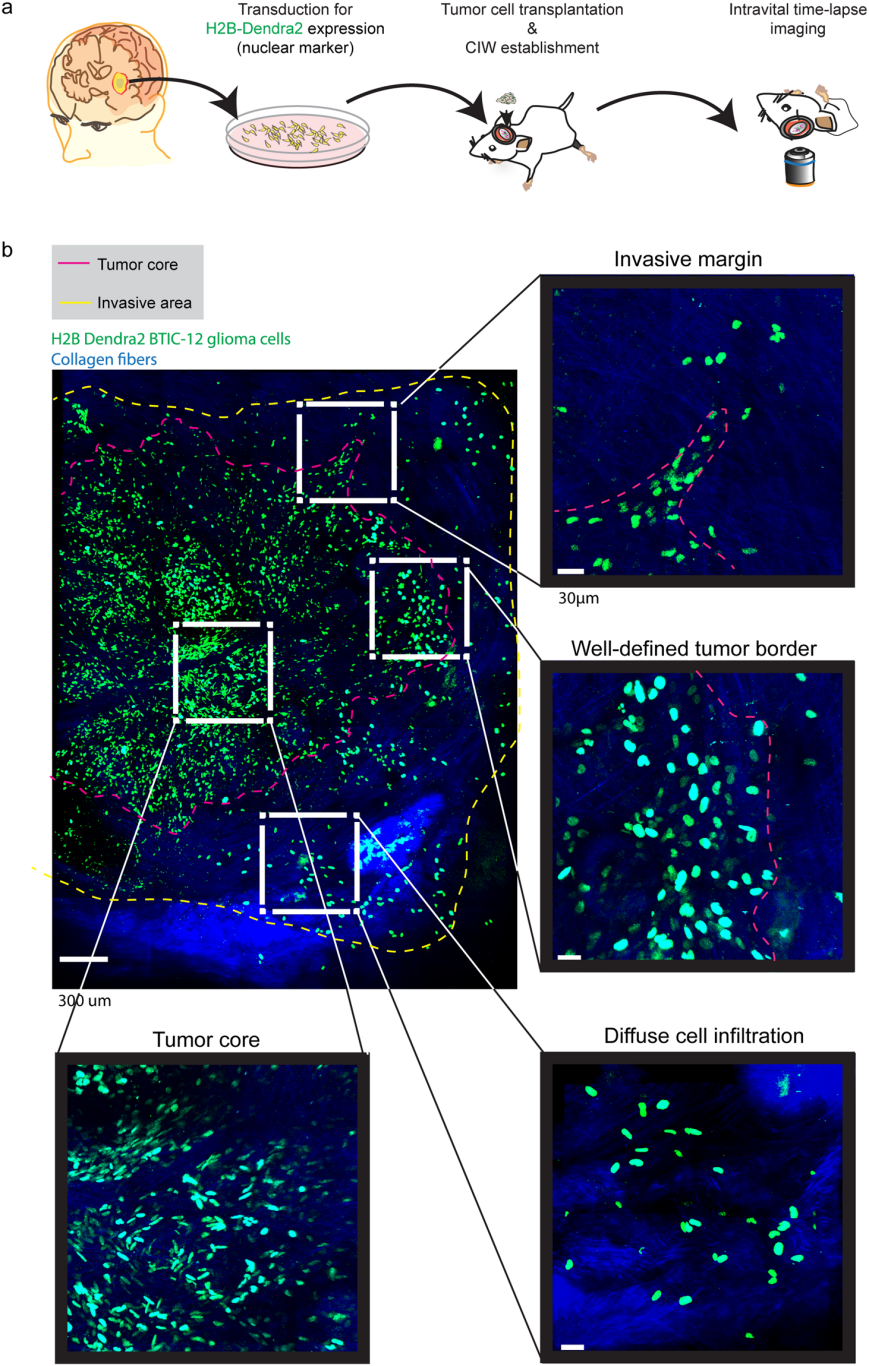
Tumor border configuration in the interphase between normal brain and tumor core. (**a**) Schematic overview of the experimental setup. Patient derived BTIC lines expressing H2B Dendra2 where implanted in the brain of NSG mice. Time-lapse *intravital* imaging was performed through a CIW to study the invasive behavior of single tumor cells. (**b**) Representative 3D reconstructed tile-scan showing distinct tumor border configurations. Shown are H2B expressing BTICs in green, collagen fibers in blue. The dotted pink line delineates the tumor core, while the dotted yellow line delineates the tumor cell invasive area. Scale bar = 300 μm.

To gain insight in glioma cells migratory behavior at different tumor border configurations we imaged the *in vivo* behavior of single BTICs derived from GBM patients who had undergone resection^15,41^. We injected two BTIC cell lines (BTIC-10 and BTIC-12) stably expressing a nuclear fluorescent protein (H2B Dendra2) in the brain of NSG mice. To gain visual access to the brain and study the invasive behavior at single cell level *in vivo*, we implanted a chronic cranial imaging window (CIW) around the injection site^35^. Upon tumor development, a series of microscopic time-lapse z-stack images of all the visible tumor volume were acquired through the CIW at multiple time points with a minimum time interval of 45 minutes (Fig. 1a). Tile-scan images revealed distinct tumor border configurations (Fig. 1b). Three different patterns of invasion were observed: protruding multicellular groups originating at the interphase between the tumor and the brain parenchyma were defined as “*invasive margin*” (Fig. 1b). Tumor margins showing no protrusions were named “*well-defined tumor border*” (Fig. 1b). Individual cell migration into the invasive area of the brain parenchyma was defined as “*diffuse cell infiltration*” (Fig. 1b). Similar types of invasion patterns can also be found in human glioblastoma biopsy samples (Supplementary Fig. S1). For comparison, cell behavior in the tumor core was evaluated (Fig. 1b). Both BTIC cells lines showed the described border configurations (Supplementary Fig. S2).

**Figure 2:**
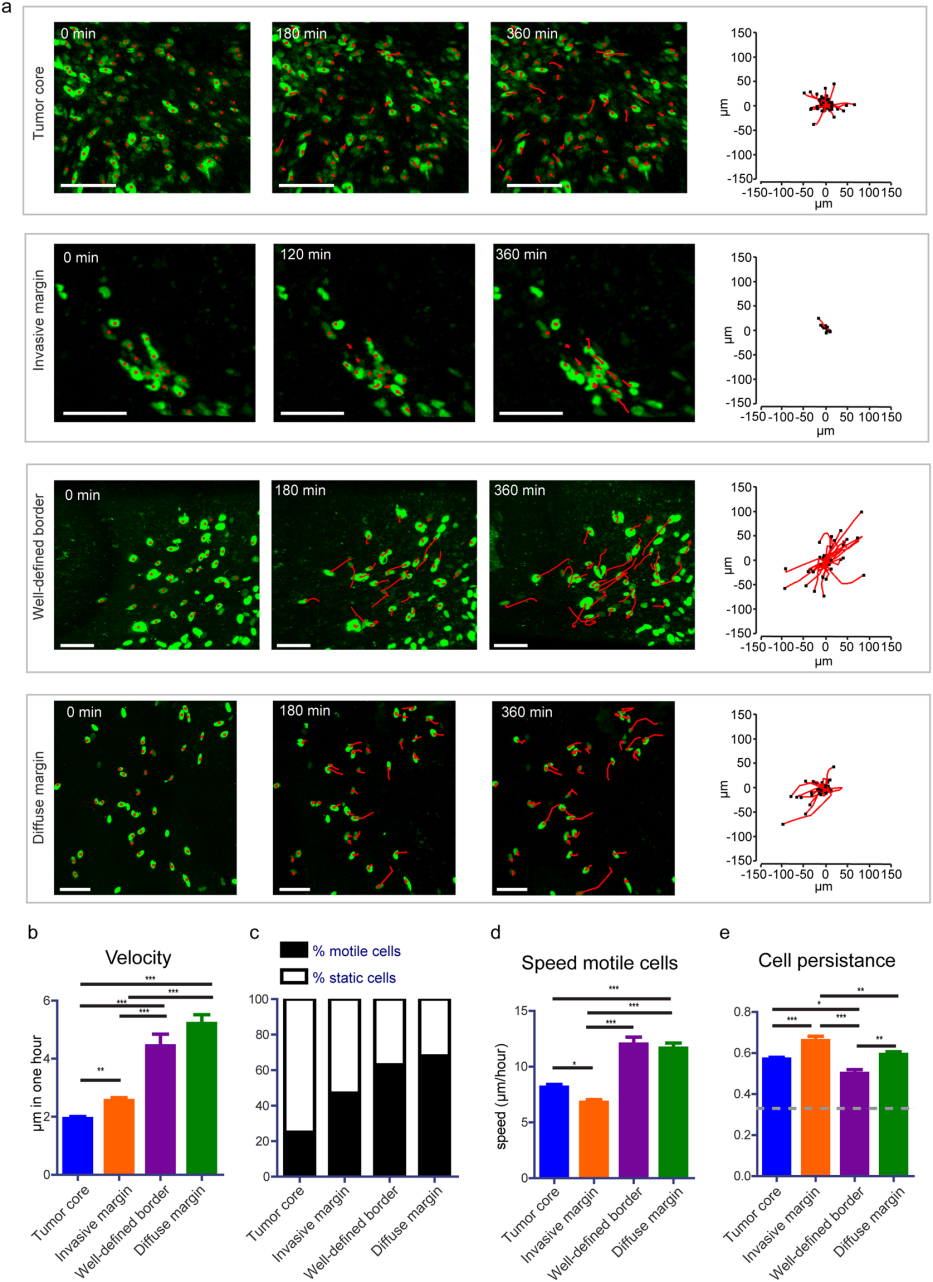
Migratory behavior of tumor cells at different border configurations. (**a**) Representative still images from a time-lapse movie showing migrating tumor cells from different border configurations. Red lines highlight individual tumor cell tracks. Scale bar = 100 μm. Corresponding plots show tracks with a common origin. (**b**) Quantification of cell velocity for the indicated border and tumor core configurations. The data is shown as mean ± S.E.M. (**c**) Percentage of motile (cell velocity > 2 μm/hour) and static cells for each condition. (**d**) Speed of motile cells at the indicated border and tumor core configurations. Data is shown as mean ± S.E.M., n = 7 mice (BTIC-10 and BTIC-12 lines). (**e**) Persistence of motile cells at the indicated border and tumor core configurations. The data is shown as mean ± S.E.M, n = 7 mice (BTIC-10 and BTIC-12 lines). *p < 0.05, **p < 0.01, ***p < 0.0001, one-way ANOVA with Tukey’s post hoc test.

The movement of individual tumor cells in distinct tumor border configurations was determined by tracking the migration path over time in 3D reconstructed time-lapse movies (Fig. 2a). Information about migration velocity, speed, persistence, and directionality was extracted from the tracks. Although there was variation in terms of cell velocity between the different mice, the relative migratory behavior between the different border configurations was consistent among them (Supplementary Fig. S2). When we performed a mixed-effects regression of tumor cell migration away from the tumor border we found that it was uncorrelated to the type of BTIC (Suppl. Table 1). Thus, we excluded that the type of BTIC had an impact on the migratory behavior and describe pooled data of both BTIC lines in further analysis.

### Role of spatial cell arrangements in migratory behavior within the *invasive margin*

**Figure 3:**
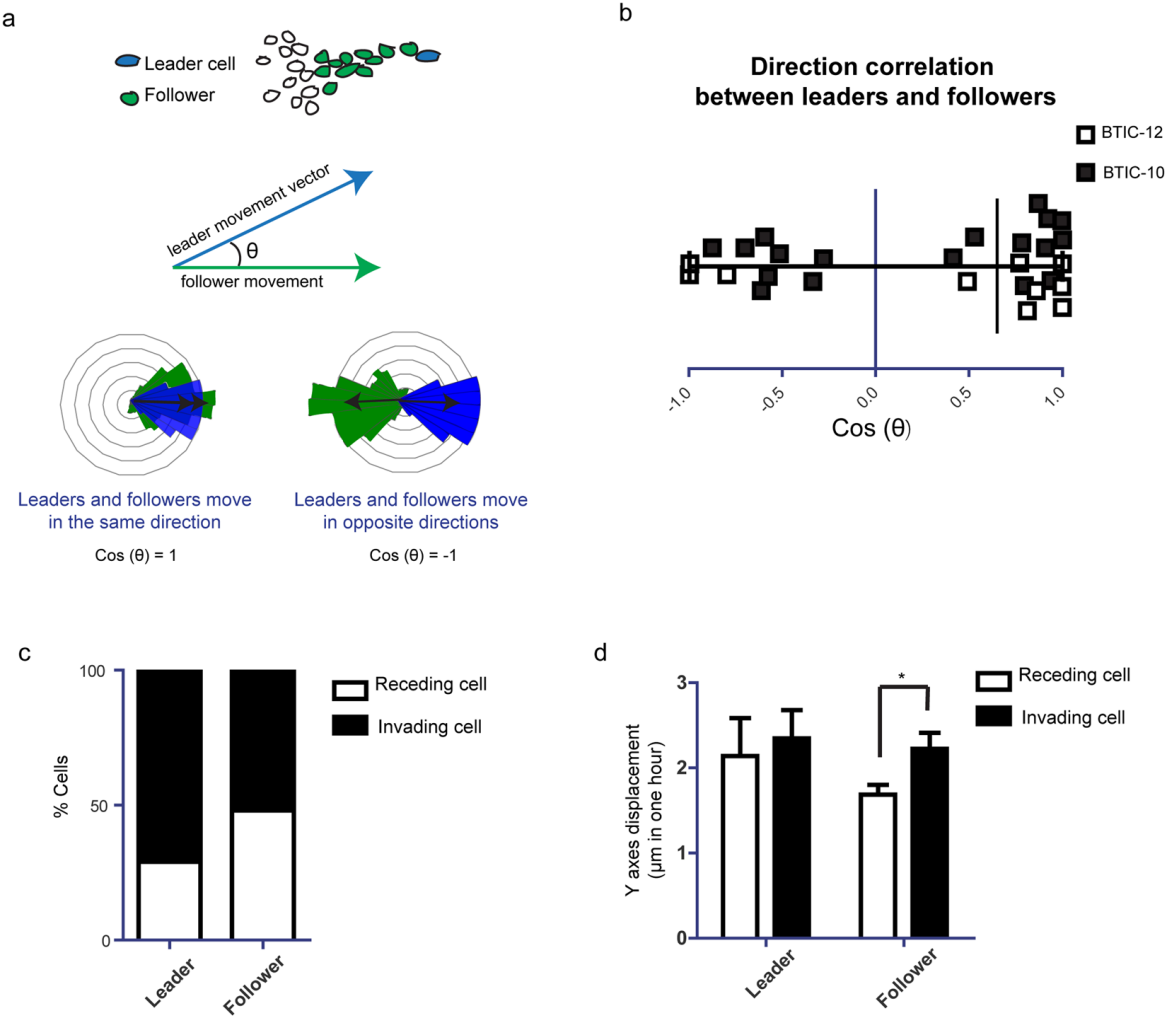
Role of spatial cell arrangements in *invasive margin* migratory behavior. (**a**) Schematic representation how the correlation of direction between leader and follower cells of the *invasive margin* was quantified. (**b**) Quantification of direction correlation between leader and follower cells. n = 7. Black squares show BTIC-10 line. White squares show BTIC-12 line. ^ns^p > 0.05, one sample Student’s *t*, hypothetical value = 0. (**c**) Percentage of invading and regressing cells for each condition, n = 7 (BTIC-10 and BTIC-12). (**d**) Quantification of Y-axes displacement for the invading/regressing cells of each condition, n = 7 (BTIC-10 and BTIC-12). *p < 0.05, Student’s *t* test. For leader and follower cells invading cells versus receding cells were tested.

Next, we aimed to understand what drives cell migration at the *invasive margin*. We hypothesized that spatial cellular arrangements at the *invasive margin* define the migration direction of subsequently following cells, as previously described^23^. Within each *invasive margin* position, we measured the direction correlation between cells leading invasion and their followers (Fig. 3a). We did not find clear correlations between the direction of movement of invasion leading cells and those following (Fig. 2b). To test the hypothesis that these data point towards a predominant role of the microenvironment to determine direction and against an individual genetic program of a subtype of cells, we re-evaluated our IVM movies and observed that cells spatially rearrange within the *invasive margin*, with leader cells becoming followers and vice versa (Supplementary Movie 1).

To further analyze spatial rearrangements, we compared the proportion of leader and follower cells moving towards and away from the tumor. We found that cells located at the leader position more often moved away from the tumor core (74%) than towards it, compared to follower cells that moved in both directions (Fig. 3c), pointing to the hypothesis that they were most strongly disposed to a microenvironmental gradient that triggers invasion^42,43^. While follower cells showed distinct velocities depending on the direction they moved, with a higher velocity when moving away from the tumor core, leader cells did not show such behavior (Fig. 3d).

Next, we analyzed invasion related to anatomical structures within the brain. We observed that BTIC use different routes of invasion along white-matter tracts or blood vessels (Supplementary Fig. 3). We further analyzed and compared the behavior of cells that used both routes of migration and found that in both cases the direction of leader cell invasion did not correlate to the direction taken by follower cells (Supplementary Fig. 4a,b,e). Perivascular leader cells had a higher tendency to move away from the tumor core than leader cells within the parenchyma (Supplementary Fig. 4c). In addition, perivascular leader cells moved faster than intraparenchymal leader cells (Supplementary Fig. 4d).

In summary, these results indicate that leader cells migrate away from the tumor core more often and faster when associated to blood vessels.

### Cell dynamics at the tumor core

To get a more holistic picture of BTIC fates within our model, we next analyzed cell migration in the non-invasive parts of the tumor (Fig. 2a). In contrast to the common believe that cells within the tumor core are static^22^, we observed that from all cancer cells in the tumor core, 23% was motile (Fig. 2c) and moved with an average speed of 8 μm/hour (Fig. 2d), a speed even higher than of the cells of the invasive front.

### Cell dynamics at the tumor border

We next analyzed the behavior of cells within the different types of tumor borders. Here, we determined the mean cell velocity of tracked single cells (cell displacement over time) and found that cells, at the *well-defined border* (4.4 µm/hour) and the *diffuse margin* (5.2 µm/hour), possessed a higher velocity than cells from the *invasive margin* (2.6 µm/hour) (Fig. 2b). Next, we compared the percentage of motile cells with a cell velocity > 2 µm/hour in between the tumor core and all kinds of borders and observed a higher proportion of motile cells in all border configurations as compared to the tumor core (Fig. 2c), pointing to a general more migratory behavior of tumor borders. The speed of motile cells (measure of the actual distance a cell covers over time) was highest at the *diffuse margin* and the *well-defined border* with 11.6 µm/hour and 12 µm/hour respectively (Fig. 2d). Cells in the *invasive margin* were found to be moving more slowly with an average speed of 6.8 µm/hour. However, when we analyzed the persistence of movement, we discovered that cells from the *invasive margin* moved in the most persistent fashion of higher than 0.36, a number that was previously considered to be representative for random walk^44^.

**Figure 4:**
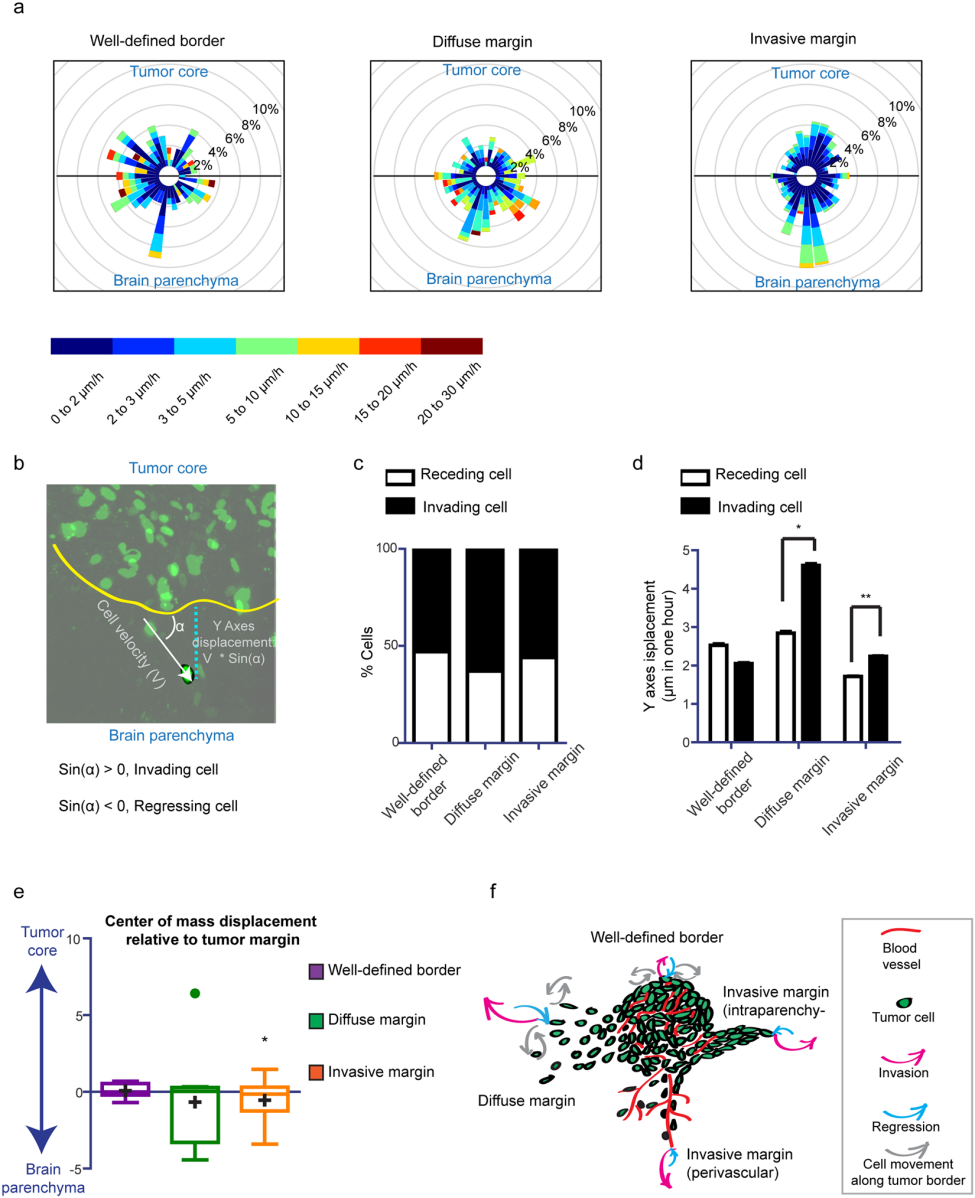
Direction of cell movement at different tumor border configurations. (**a**) Wind rose plot representing the percentage of cells migrating in the direction of a leaflet for each tumor border configuration. Color scale indicates migration speed, n = 7 (BTIC-10 and BTIC-12). (**b**) Schematic representation of Y-axes cell displacement quantification. (**c**) Percentage of invading and regressing cells for each condition, n = 7 (BTIC-10 and BTIC-12). (**d**) Quantification of the Y-axes velocity for invading and regressing cells of each condition, respectively, n = 7 (BTIC-10 and BTIC-12), *p < 0.05, Student’s *t* test. For each border configuration invading cells versus regressing cells were tested. (**e**) Tukey-style whiskers plot of the center of mass displacement of individual positions of each condition. Boxes indicate the interquartile range, crosses indicate the mean, and dots indicate outliers. n = 7 (BTIC-10 and BTIC-12), *p < 0.05, one sample Student’s *t* test, hypothetical value = 0. When the center of mass does not change in respect to its initial position it is considered to be 0. The one sample Student´s t test determines if the mean of the center of mass displacement (COMd) of the positions belonging to the distinct border types is different from 0. COMd > 0: overall the cells in this type of position move towards the tumor core. COMd < 0: overall the cells in this type of position move away from the tumor core. (**f**) Schematic illustration of the model showing cell dynamics at different tumor border configurations.

Since directionality is an important feature of invasion, we analyzed the directionality patterns in each type of border configuration. We observed disperse directionality patterns in all subtypes (Supplementary Fig. S5), with cells migrating towards the parenchyma, while other cells migrated towards the tumor core, or parallel to the tumor border (Fig. 2a, Supplementary Movie 1). Moreover, we observed cells that changed migration directionality over time (Fig. 2a, Supplementary Movie 1). To assess whether a particular migration behavior is favored within one of the border configurations, we plotted the migration path of all the cells from different positions in a wind rose plot (Fig. 4a). Here, we found the most directed and invasive pattern in the *invasive margin* configuration.

To identify the velocity and proportion of phenotypically relevant invading and retreating cells, we measured the perpendicular cell displacement relative to the tumor margin (Fig. 4b). We found that the proportion of invasion was balanced at all border configurations with only a slight prevalence of invading cells (Fig. 4c). The *diffuse margin* harbored the highest proportion of invading cells (61.5%), followed by the *invasive margin* (54.4%) (Fig. 4c). Moreover, when we compared the velocity of the invading and retreating cells we found that invading cells moved faster than retreating cells at both the *invasive margin* and the *diffuse margin*, but not at the *well-defined border* (Fig. 4d).

Finally, to depict if all areas within a specific border configuration follow the same phenotypic behavior, we assessed the overall displacement of the center of mass perpendicular to the tumor margin (COMy) (Fig. 4e). In all border configurations, we found positions moving towards and away from the tumor border (Fig. 4e), indicating that not all the regions of a tumor are in continuous expansion. Our results showed that for the *well-defined border* configuration the mean COMy was close to zero, indicating that this type of configuration, although very dynamic within the position (Fig. 2a), does not contribute to cell spreading away from the tumor. However, both the *diffuse* and *invasive margin* showed a mean COMy shifted towards the brain parenchyma (Fig. 4e), emphasizing that these configurations may drive tumor cell spreading away from the tumor.

To further confirm this observation, we used a multivariate linear model to more accurately evaluate the association of COMy with factors such as mean velocity of invading cells, mean velocity of retreating cells and their interaction with variables such as frequency of invading cells, configuration type and BTIC type (Suppl. Table 2). Our model shows that for each position the velocity of invading cells was the strongest predictor of tumor expansion (COMy shift towards the brain parenchyma) and was independent of the BTIC cell line. The velocity of invading cells showed interaction effects with two other predictors (Supplementary Fig. 6), namely frequency of invading cells and border configuration type. We consolidated variation to these predictors: with a high proportion of invading cells, the velocity of invading cell has a higher impact on tumor expansion (COMy); the velocity of invading cells has a higher impact on cell spreading and at the *invasive margin* and at the diffuse border (Supplementary Fig. 6a).

Since our analysis showed that cells at the *invasive margin* have a lower cell velocity (Figs 2b and 4d), this factor alone could not explain a similar effect on cell spreading at the *invasive margin* and at the *diffuse border* as shown in Fig. 4e. Therefore, we analyzed the distribution of the frequency with which cells were invading within each position for each configuration type and found that the *invasive margin* showed more positions with a high frequency of invading cells compared to the *diffuse margin* (Supplementary Fig. 6b).

Combined, these data indicate that the *diffuse margin* and *invasive margin* contribute to cell spreading and tumor expansion through two different mechanisms: a higher speed for the first, and a higher proportion of invading cells for the second.

## Discussion

In contrast to glioblastoma morphology, tumor cell dynamics within GBM is not well understood. We combined an orthotopic GBM model, time-lapse *in vivo* microscopy of human brain tumor initiating cells through a cranial window, and in-depth analysis of tumor cell spreading to acquire a better insight of GBM tumor cells dynamics within their environment on multiple layers.

Using this approach, we delineated tumor border configurations conformed by two different types of invasion represented by cells in the *invasive margin* and *diffuse infiltration* pattern; and one pattern that, although dynamic, seems to be non-invasive, the *well-defined tumor border* pattern. We discovered that, in contrast to the other patterns^45^, cells from the *invasive margin* moved with lower velocity and speed, but in the most persistent fashion. At the *diffuse margin* cells moved less persistently but also contributed to cell spreading and tumor expansion through a higher speed of migration. Moreover, we found that not all regions that show tumor cell migration actually contribute to tumor cell invasion. GBM cells in the tumor *well defined border*, a region generally thought to be static^22^, are also extremely dynamic, but do not contribute to invasion as their movement shows a highly undirected pattern. This highlights the importance of this study, indicating that a more comprehensive analysis of GBM including morphological and dynamic data may allow deeper insights in the complex mechanism of invasion. In contrast, studies that looked solely on tumor cell migration speed, or were performed *in vitro*, models where tumor cells lack microenvironmental cues for directed migration, do not fully elucidate this heterogeneity^30,46^.

Glioma cells have previously been reported to use different routes of invasion such as intraparenchymal invasion along white-matter tracks^16,21^ or invasion along blood vessels^16^. In line with this, in our model, perivascular leader cells had also a higher tendency to move away from the tumor core than cells within the parenchyma, and to move faster. These data correspond well to published data, where individually migrating cells, collective strands extending along blood vessels or white matter tracts, and multicellular networks of interconnected glioma cells were found^22,45,47–49^. However, this is the first piece of work that analyzed single-cell dynamics of patient derived tumor cells *in vivo* at different tumor border and invasive front areas corresponding to the morphologies found in patients.

It has been previously described that collectively moving cells can either have a specific role defined by their position in the cellular stream, or dynamically change position with any cell from the group with the ability to drive migration^23^. Against our expectation, we did not find clear correlations between the direction of movement of invasion leading cells and those following. Our results show that in our model tumor cells can dynamically exchange position in the collective cell stream indicating that their behavior may be more dependent of microenvironmental influences than of genetic predisposition. Cells located at the leader position more often moved away from the tumor core than towards it, compared to follower cells that moved in both directions. This possibly points to the hypothesis that leader cells were most strongly disposed to a microenvironmental gradient that triggers invasion^50–52^, probably due to distinct microenvironmental structures^45,53^ or gradients of chemoattractants^50^. Moreover, since we found migratory cells in all areas of the tumor, it is likely that all cells have an intrinsic capacity to migrate, however only some actually contribute to invasion due to microenvironmental cues.

The fact that tumor cells move bidirectionally indicates that tumor cells may receive chemotactic signals from both the tumor core and the tumor microenvironment. Indeed, studies based on brain slices engrafted with tumor spheres showed a cell fraction with a highly invasive morphology that interacted with the tumor core^54–56^. In addition, *ex vivo* and *in vivo* studies using IVM have also shown that tumor cells that are integrated into functional connective networks respond to tumor core removal or injury by repopulating the injured area^54,57^. We have also found that tumor biopsy-like injury changes tumor cell migratory capacity and increases tumor cell migration speed^31^, all contributing to the hypothesis that invasion is highly dependent on the environment. Another potential driver of glioma tumor cell migration is the direct movement of stromal cells. Although there is no evidence so far that that brain parenchymal cells can directly drive tumor cell migration, in squamous cell carcinoma, fibroblasts have shown to lead collective migration of tumor cells^58^. Moreover immune cells, such as macrophages, have shown to associate with breast tumor cells, drive their migration towards blood vessels and facilitate their intravasation^59^. The possibility of parenchymal brain cells such as e.g. microglia as direct drivers of glioma cell migration still needs to be explored.

In summary our work is the first analytical study that correlates distinct tumor border patterns that can be found on histological sections with particular tumor cell dynamics. It not only fosters the understanding of single cell invasion, but possibly also of distant metastasis and re-population of the primary tumor site, both mechanisms that significantly influence the prognosis of patients with GBM. Our long-term goal is to correlate the analysis of tumor morphology with the dynamics of tumor cell movement to get a more complete picture of GBM invasion and to therefore improve the prognostic evaluation of GBM and to possibly develop novel therapeutic approaches. Future work should be directed to analyze invasive behavior of a much larger cohort of distinct patient-derived tumor cell lines, in order to confirm our results and to allow correlations with morphological and possibly prognostic features. Since it is known that the adaptive immune system can also influence tumor migratory behavior^18,60^, it might be worth to use humanized tumor mouse models to assess the role of the human immune microenvironment on tumor cell behavior.

## Materials and Methods

### Tumor cell lines

We used previously established primary brain tumor initiating cell lines (BTIC)-10 and -12 derived from patients with IDH wild type glioblastoma as described^15,41^. The Department of Neuropathology, University of Regensburg (MJR), verified the patients’ diagnoses and WHO grade.

Tumor cells were maintained in RHB-A (Y40001, Takara), supplemented with 20 ng/ml of EGF (130097751), bFGF (130093842) (both Miltenyi Biotech), and 50 U (v/v) Penicillin/0.05% (v/v) Streptomycin (P4333) (Sigma-Aldrich) at 37 °C, 5% CO_2_, 95% humidity in a standard tissue culture incubator. Progenitor features of BTICs were verified by clonogenicity assays, flow cytometry (CD133, CD15, CD44, A2B5), immunocytochemistry (Nestin, Sox2, GFAP), and tumor take in an immunocompromised mouse model (female NOD.Cg-Prkdcscid Il2rgtm1Wjl/SzJ). BTICs were lentivirally transduced to achieve stable expression of the nuclear fluorescent protein H2B Dendra2, as described^61^.

The ethics board of the University of Regensburg, Germany, approved the use of human material for this study (No° 11-103-0182) and all patients gave written informed consent. All methods were performed in accordance with the relevant guidelines and regulations.

### Animals

Non-obese diabetic SCID IL-2 receptor gamma chain knockout (NSG) mice 8 to 12 weeks *post partum* were used for the experiments. Mice were housed in an individually ventilated cage and received food and water *ad libitum*. All experiments were carried out in accordance with the guidelines of the Animal Welfare Committee of the Royal Netherlands Academy of Arts and Sciences, the Netherlands. The experimental protocols used in this manuscript were approved by the Centrale Commissie Dierproeven (CCD) and the Instantie voor Dierenwelzijn (IvD).

### Cranial imaging window (CIW) surgery and tumor cell injection

CIW surgery and tumor cell injection were performed at the same day as described^31,62^. Briefly, mice were sedated with Hypnorm (Fluanison [neuroleptic] + Fentanyl [opioid]) (0.4 ml/kg) + Midazolam [benzodiazepine sedative] (2 mg/kg) at a dose of 1:1:2 in sterile water and mounted in a stereotactic frame. The head was secured using a nose clamp and two ear bars. The head was shaved and the skin was cut in a circular manner. The mouse was placed under a stereo-microscope to ensure precise surgical manipulation. The periosteum was scraped and a circular groove of 5 mm diameter was drilled over the right parietal bone. The bone flap was lifted under a drop of cortex buffer (125 mM NaCl, 5 mM KCl, 10 mM glucose, 10 mM HEPES buffer, 2 mM MgSO4 and 2 mM CaCl2, pH 7.4) and the dura mater was removed. Gelfoam sponge (Pfizer) was used to stop bleeding. (Supplementary Fig. 7).

Next, 1 × 10^5^ BTIC H2B-Dendra2 cells suspended in 3 μl of PBS were injected stereotactically using a 10 μl Hamilton syringe with a 2 pt style in the middle of the craniotomy at a depth of 0.5 mm. The exposed brain was sealed with silicone oil and a 6 mm coverslip glued on top. Dental acrylic cement (Vertex) was applied on the skull surface, covering the edge of the coverslip and a stainless steel ring was glued around the coverslip to provide fixation to the microscope. A single dose of 100 μg/kg of buprenorphine (Temgesic, BD pharmaceutical limited) was administered. Mice were closely monitored twice per week for behavior, reactivity and appearance.

### Intravital imaging

Mice were imaged as previously described^31^. In short, mice were sedated and placed face-up in a custom-designed imaging box. Time-lapse images of the entire tumor volume were acquired for a maximum of 13 hours. The minimal time interval between serial images was set to 45 minutes. For tile-scans, images of the complete z-stack of the tumor were acquired to a depth of 300 μm, with a step size of 3 µm (typically 70–100 images in each z-stack). In a group of 4 mice, blood vessels were imaged by intravenous injection of 70 kDa Dextran–Texas Red (Invitrogen Life Technologies) or 2.000.000 kDa Dextran-Rhodamine (Thermofisher scientific).

Imaging was performed on an inverted Leica SP8 multiphoton microscope with a chameleon Vision-S (Coherent Inc., Santa Clare, CA, www.coherent.com), equipped with a 25 x water objective (HCX IRAPO NA0.95 WD 2.5 mm) with four HyD detectors: HyD1 (560–650 nm), HyD2 (500–550 nm), HyD3 (455–490 nm), and HyD4 (<455 nm). Images were acquired at a wavelength of 960 nm, where H2B Dendra2 as well as both Dextran reagents showed sufficient signal intensity. Collagen (second harmonic generation) was detected using HyD4, H2B-Dendra2 was detected with HyD2 and Dextran reagents were detected with HyD1. Scanning was performed in a bidirectional mode at 400 Hz and 12 bit, with a zoom of 1 × , 512 × 512 pixels.

### Image processing and analysis

For 3D visualization, shift correction, rendering and data analysis time-lapse movies were processed with Imaris (Bitplane, Switzerland). The Spot Analysis module was used for semi-automated tracking of cell motility in three dimensions and for shift correction. Data containing the coordinates of each cell, the values of cell direction, speed (per time unit) and velocity (the vector of movement) was exported and processed and plotted in GraphPad or R. As the animal’s pulse and breathing can cause motion artifacts, we decided that only cells with a velocity (displacement) more than 2.0 µm/hour were classified as motile.

Regions with distinct patterns of invasion were defined as described above by visual assessment of the first frame of the movies. Only regions with a clear invasive pattern were included in the analysis.

The windrose plot representing cell direction and speed was created using R package “openair”^63^. To measure the individual cell and center of mass displacement perpendicular to the tumor border the ‘Chemotaxis and Migration Tool’ (Ibidi GmbH) was used^64^. The results of Imaris tracking were converted in 2D along the *x* and *y* axes and directly imported into the ‘Chemotaxis and Migration Tool’ software tool. The cell trajectories were all extrapolated to (x, y) = 0 at time 0 h (Fig. 2a) to visualize the trajectories of each cell with a common origin. Next the cell trajectories were rotated for each position using “Data rotation” tab of the software in order to get the tumor margin parallel to the x axis (as in Fig. 4b). The tumor margin was defined as the tangent drawn to the main tumor mass. For each cell trajectory the angle of cell displacement vector in respect to the x axes (tumor border) was extracted using the ‘Chemotaxis and Migration Tool’. Finally individual cell displacement perpendicular to the tumor border was measured as:

$${Y}_{axesdisplacement}=Cell\,velocity\times \,\sin (angl{e}_{relativetotumorboder})$$

The spatial average of all cell positions was used to measure the center of mass displacement perpendicular to the tumor border. For each position the difference in the center of mass along the Y axes between its initial value and that at the end of the experiment was measured by means of the ‘Chemotaxis and Migration Tool’. This measure represented per unit of time was termed the displacement of center of mass (COMy), where n is the number of cells per individual position:

$${M}_{start}=\frac{1}{n}{\sum }_{i=1}^{n}({y}_{i,start})=(0);{M}_{end}=\frac{1}{n}{\sum }_{i=1}^{n}({y}_{i,end})$$

$$COMy=\frac{{M}_{end}-{M}_{start}\,}{hour}$$

3D tile scan projections were used for illustration of different border configurations. For illustration of tumor cell migration, 3D images were tracked manually with an ImageJ plugin (“MTrackJ” Rasband, W.S., ImageJ, U.S. NIH, Bethesda, Maryland, USA).

The identities of leader and follower cells were defined at the first frame of the time-lapse and were maintained through all the movie. The first cells protruding from an *invasive margin* morphology were defined as “leader”. All the consequent cells from the *invasive margin* were defined as “follower”. The direction correlation between leader and follower cells of the *invasive margin* was calculated as the cosine of the angle of the leader cell and all the follower cell paths (Fig. 3a). Values close to 1 indicate correlation of direction, while values close to −1 indicate opposite direction correlation.

As i.v. injected Dextran reagents leak out of the vessels in the course of time, blood vessel structure from the first time point was applied to all time points for illustration of the blood vessels in the time-lapse stills.

### Patient glioblastoma samples

Archives of the Department of Neuropathology, University Hospital Regensburg, were reviewed for glioblastoma cases sampled with parts of the infiltrative rim and representing a spectrum of infiltration patterns observed in the mouse glioblastoma window model. Immunohistochemical staining was performed in context of the routine diagnostic work-up of the samples following a standard protocol^65^. Antibodies used were as follows: GFAP (Clone 6F2), Dako #M0761, 1:200 dilution; p53(Clone BP53-12) Santa Cruz Biotechnology #SC-263, 1:2000 dilution; Ki-67(Clone MIB-1) Dako #M7240, 1:200 dilution.

### Statistical analysis

For all normally distributed measurements, the Student’s *t* test (comparison of two mean values) or one-way ANOVA (when >2 means were compared) were used to determine significance, set to p < 0.05. Post-hoc tests were performed with p values < 0.05. All p values were two-tailed. Levels of significance were set as follows: ^ns^p > 0.05, *0.05 ≤ p > 0.01, **0.01 ≤ p > 0.001, ***0.001 ≤ p > 0.0001, ****p ≤ 0.0001. Error bars are presented as ± S.E.M. All statistical analysis were performed using GraphPad Prism software (version 6, GraphPad Software, USA).

## Supplementary information

Suppl. material

Suppl. video 1

## Data Availability

The authors declare that all data supporting the findings of this study are available within the article and its Supplementary Information files, or from the corresponding author (MA) upon request.
