# Supplementary material for: Intravital imaging of glioma border morphology reveals distinctive cellular dynamics and contribution to tumor cell invasion
Source: Sci Rep. 2019 Feb 14;9:2054. doi: 10.1038/s41598-019-38625-4 (PMC6375955; doi:10.1038/s41598-019-38625-4)
Supplement: Supplementary file 1 — Suppl. material [file 41598_2019_38625_MOESM1_ESM.pdf]

Title

Intravital imaging of glioma border morphology reveals distinctive cellular dynamics and contribution to tumor cell invasion

Authors

Maria Alieva [1,2*#], Verena Leidgens [1,3#], Markus J. Riemenschneider [4], Christoph A. Klein [5], Peter Hau [3*$] and Jacco van Rheenen [1,6$]

Supplementary Figures

Supplementary Fig 1 Tumor infiltration patterns in human glioblastoma samples. Right column shows glioblastoma with typically *diffuse cell infiltration* in the surrounding brain tissue (right column). Note that there is a decreasing gradient in cell density from the upper right to the lower left of the images. Captured is a cortical infiltration zone (**CI**) with interspersed neurons. GFAP staining in the illustrated case hardly differentiates between infiltrative tumor cells and reactive astrocytes. A relevant number of tumor nuclei stain for the p53 tumor suppressor protein. Single tumor cell infiltration in the lower left of the image (**CI**) is highlighted by staining with the proliferation marker Ki-67 (MIB-1). Left column shows a tumor with a more circumscribed growth pattern with an appearance resembling the *well-defined tumor boarder* pattern in the model (red arrow). In the illustrated case solid tumor tissue (**T**) and surrounding non-neoplastic brain tissue (**B**) can be clearly differentiated. This is obvious also in the immunohistochemical stains with reactive GFAP expression and virtually absent p53 and MIB-1 labeling in the brain tissue (**B**). Note that the MIB-1 stain, however, highlights finger-shaped protrusions (black arrow) into the surrounding non-neoplastic tissue corresponding to the *invasive margin* pattern in the model. The finger-shaped protrusions can be also identified in the corresponding H&E image.

Supplementary Fig 2 Velocity of different border configurations in individual mice. Quantification of cell velocity at indicated border and tumor core configurations. Data from individual mice and BTIC-10 and -12 is plotted in separate graphs. The data is shown as mean ± S.E.M.

Supplementary Fig 3 Intraparenchymal and invasive tumor margin.

Representative 3D reconstructed tile-scan showing perivascular and intraparenchymal *invasive margin*. Shown are H2B expressing BTICs in green, collagen fibers in blue, blood vessels in red. Scale bar = 300 μm.

Supplementary Fig 4 Comparison between the intraparenchymal and invasive tumor margin.

(A) Quantification of cell velocity, percentage of motile (cell velocity > 2 μm/hour) and static cells, speed of motile cells and persistence of motile cells for the the intraparenchymal and invasive tumor margin. Data is shown as mean ± S.E.M., n = 4 mice (BTIC-10 and BTIC-12 lines). The data is shown as mean ± S.E.M, n = 4 mice (BTIC-10 and BTIC-12 lines). $*p < 0.05$, $**p < 0.01$, $***p < 0.0001$, one-way ANOVA with Tukey's post hoc test. B) Representative still images from a time-lapse movie showing migrating tumor cells from perivascular and intraparenchymal *invasive margin*. White lines highlight individual tumor cell tracks. Scale bar = 30 μm. (C) Percentage of invading and receding cells for each condition. (D) Quantification of the Y-axes displacement for invading and receding cells of each condition. n = 7 (BTIC-10 and BTIC-12). (E) Quantification of direction correlation between perivascular and intraparenchymal *invasive margin*. n = 4. $^{ns}p > 0.05$. One sample Student's *t*, hypothetical value = 0.

Supplementary Fig 5 Direction of cell migration at different positions.

Representative wind rose plots showing distinct direction of cell migration at individual positions from the different border configurations. Direction of a leaflet represents the percentage of cells migrating in that direction. Color scale indicates migration speed.

Supplementary Fig 6 Linear regression model for cell spreading into the parenchyma. (A) Linear regression model for cell spreading with the corresponding coefficients for each border configuration type. Variables are explained in Table2. (B) Histograms showing the frequency distribution of the incidence of invasion within a position for the *invasive margin* and the *diffuse margin.*

Supplementary Fig 7 Intracranial imaging window

Descriptive image showing the cranial imaging window in the living mouse in overview and magnification.

Supplementary tables

Supplementary Table 1 Summary of Mixed-effect Linear Regression Model for cell spreading perpendicular to the tumor border

Supplementary Table 2 List of simulation parameters

Supplementary movie

Supplementary Movie 1 Invasive front cell dynamics.

Intravital imaging video showing BTIC-10 H2B-Dendra2 cells migrating in opposite directions at the *invasive margin*. Colored arrows follow individual cells of the *invasive margin*. Top: tumor core; bottom: brain parenchyma. Scale bar represents 30 µm.

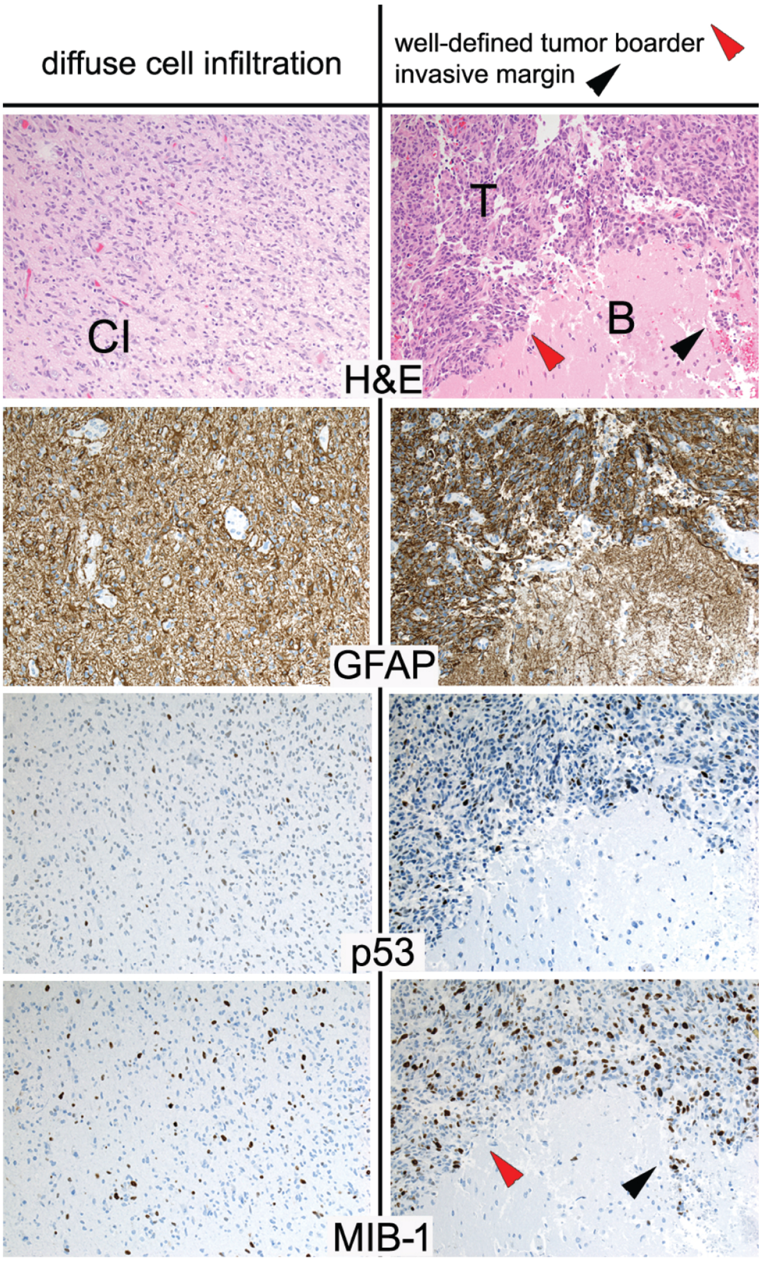

diffuse cell infiltration
well-defined tumor boarder
invasive margin
CI
T
B
H&E
GFAP
p53
MIB-1

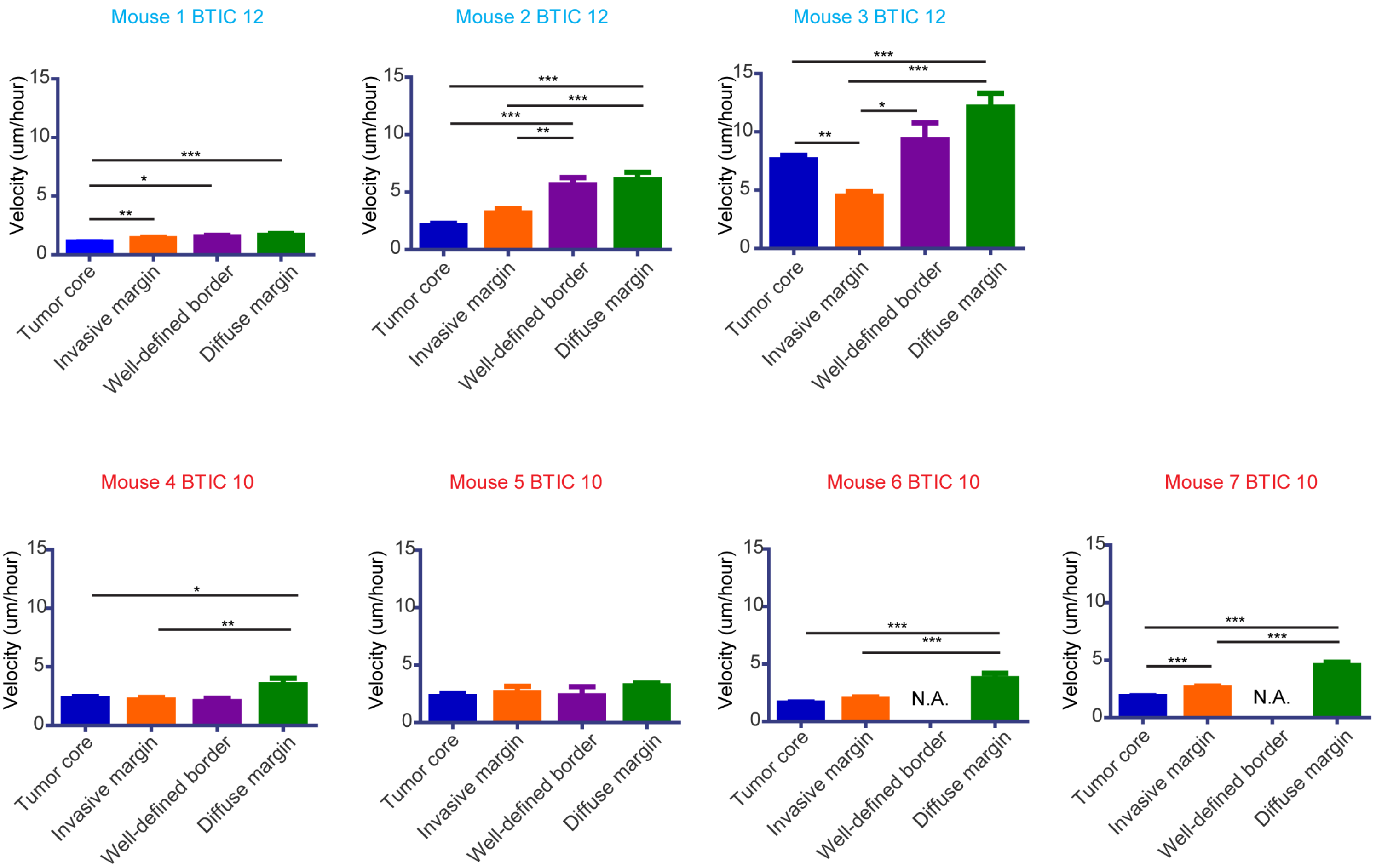
Mouse 1 BTIC 12
Mouse 2 BTIC 12
Mouse 3 BTIC 12
Mouse 4 BTIC 10
Mouse 5 BTIC 10
Mouse 6 BTIC 10
Mouse 7 BTIC 10
Velocity (um/hour)
0
5
10
15
Tumor core
Invasive margin
Well-defined border
Diffuse margin
N.A.
*
**
***

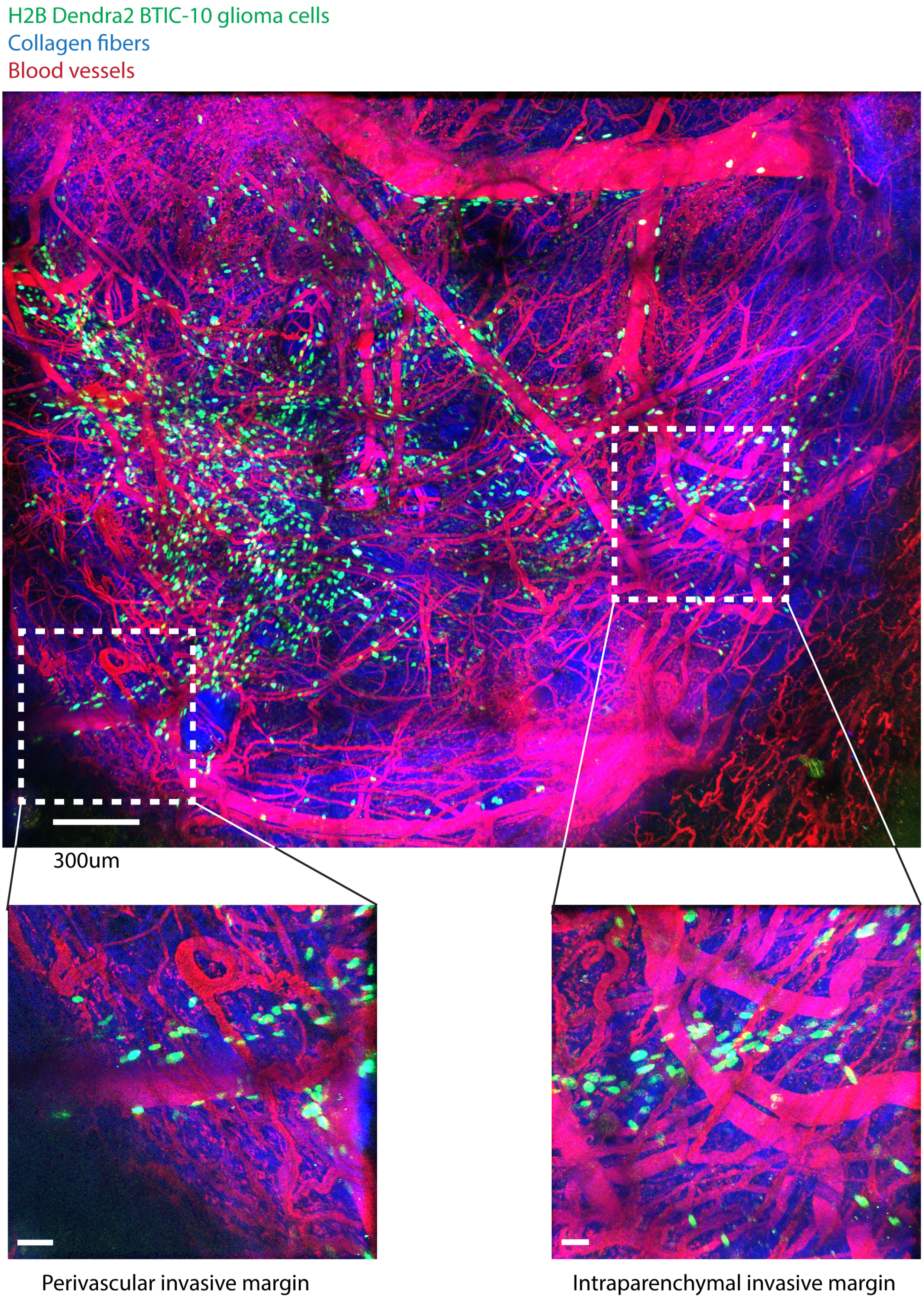
H2B Dendra2 BTIC-10 glioma cells
Collagen fibers
Blood vessels
300um
Perivascular invasive margin
Intraparenchymal invasive margin

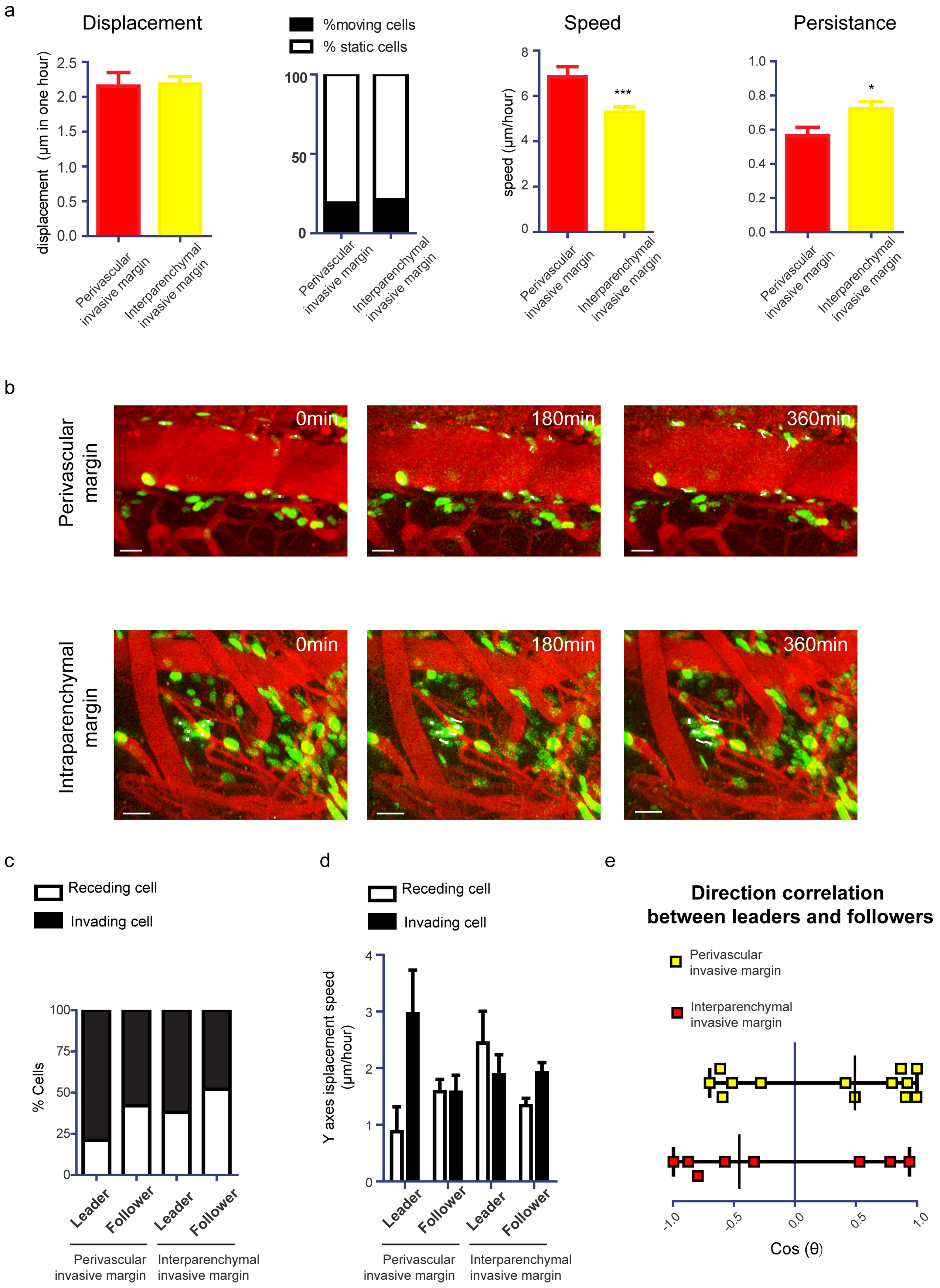
a
Displacement
displacement (µm in one hour)
2.5
2.0
1.5
1.0
0.5
0.0
Perivascular invasive margin
Interparenchymal invasive margin
%moving cells
% static cells
100
50
0
Speed
speed (µm/hour)
8
6
4
2
0
***
Persistance
1.0
0.8
0.6
0.4
0.2
0.0
*
b
Perivascular margin
Intraparenchymal margin
0min
180min
360min
c
Receding cell
Invading cell
% Cells
100
75
50
25
0
Leader
Follower
Perivascular invasive margin
Interparenchymal invasive margin
d
Y axes isplacement speed (µm/hour)
4
3
2
1
0
e
Direction correlation between leaders and followers
Perivascular invasive margin
Interparenchymal invasive margin
-1.0
-0.5
0.0
0.5
1.0
Cos (θ)

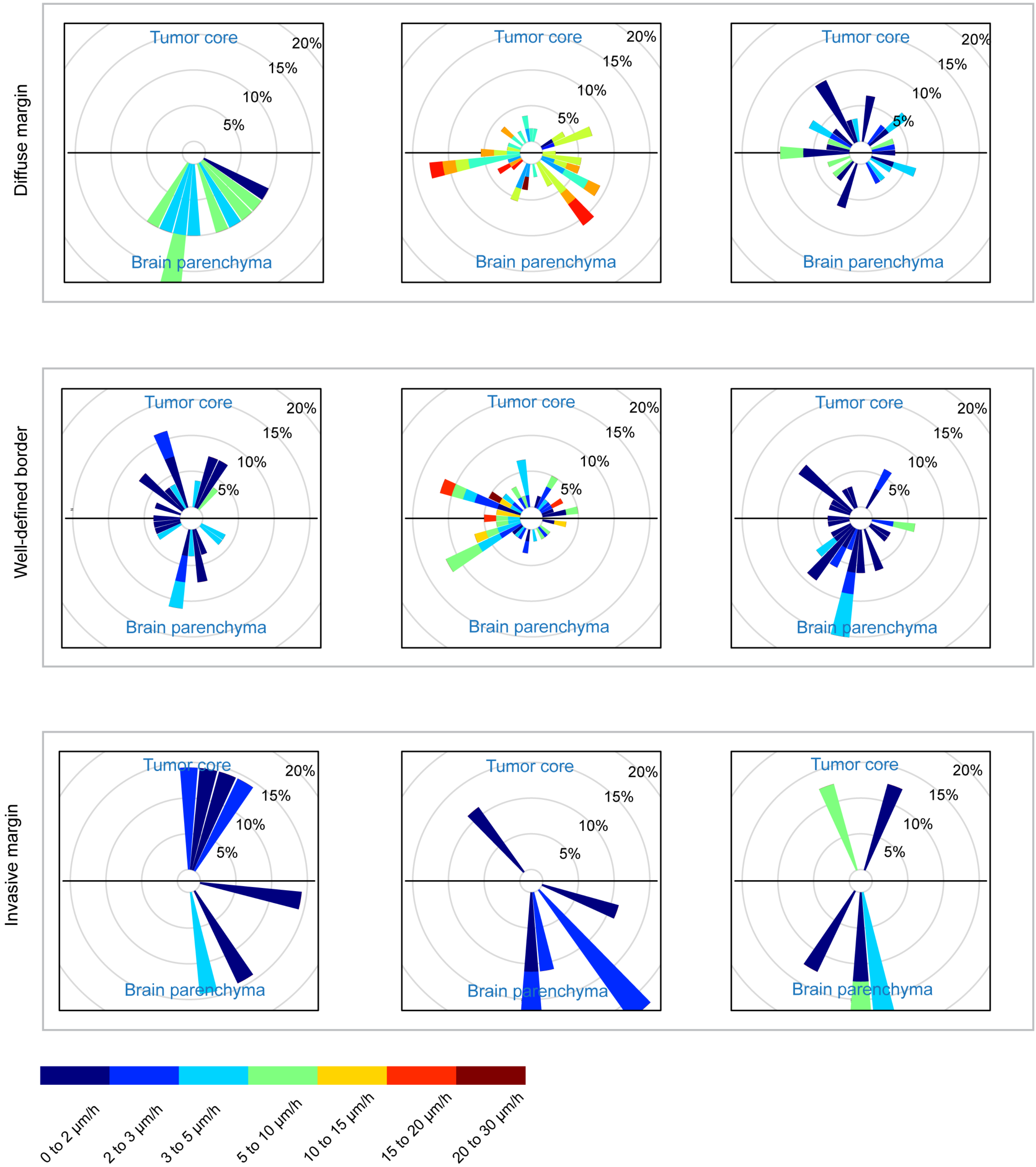
Diffuse margin
Well-defined border
Invasive margin
Tumor core
Brain parenchyma
20%
15%
10%
5%
0 to 2 µm/h
2 to 3 µm/h
3 to 5 µm/h
5 to 10 µm/h
10 to 15 µm/h
15 to 20 µm/h
20 to 30 µm/h

a

$$\text{COM } y_{border} = 0.36 + 0.86\,Vinv - 1.69(Vinv \times Finv)$$

$$\text{COM } y_{invasive} = 0.36 + 0.62\,Vinv - 1.69(Vinv \times Finv)$$

$$\text{COM } y_{diffuse} = 0.36 + 0.64\,Vinv - 1.69(Vinv \times Finv)$$

$R^2 = 0.933$
$P < 0.001$

b

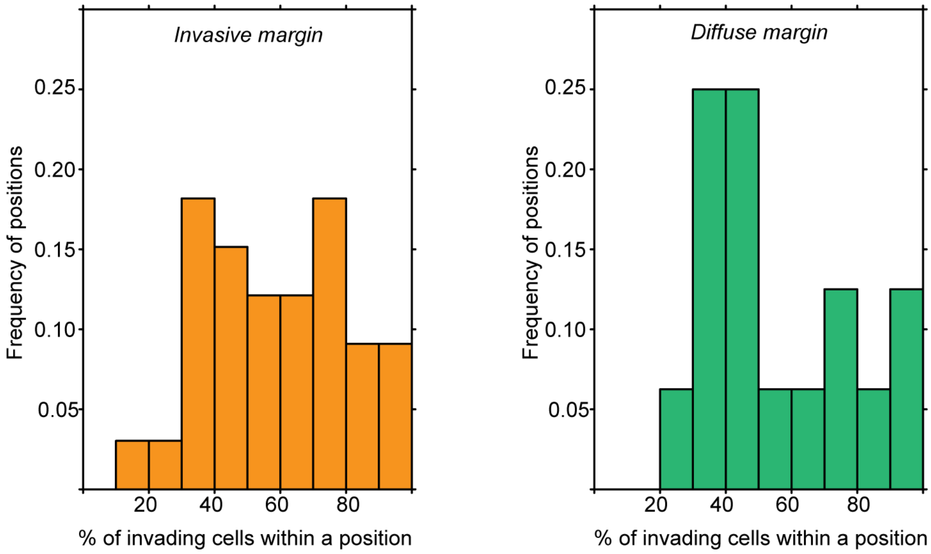

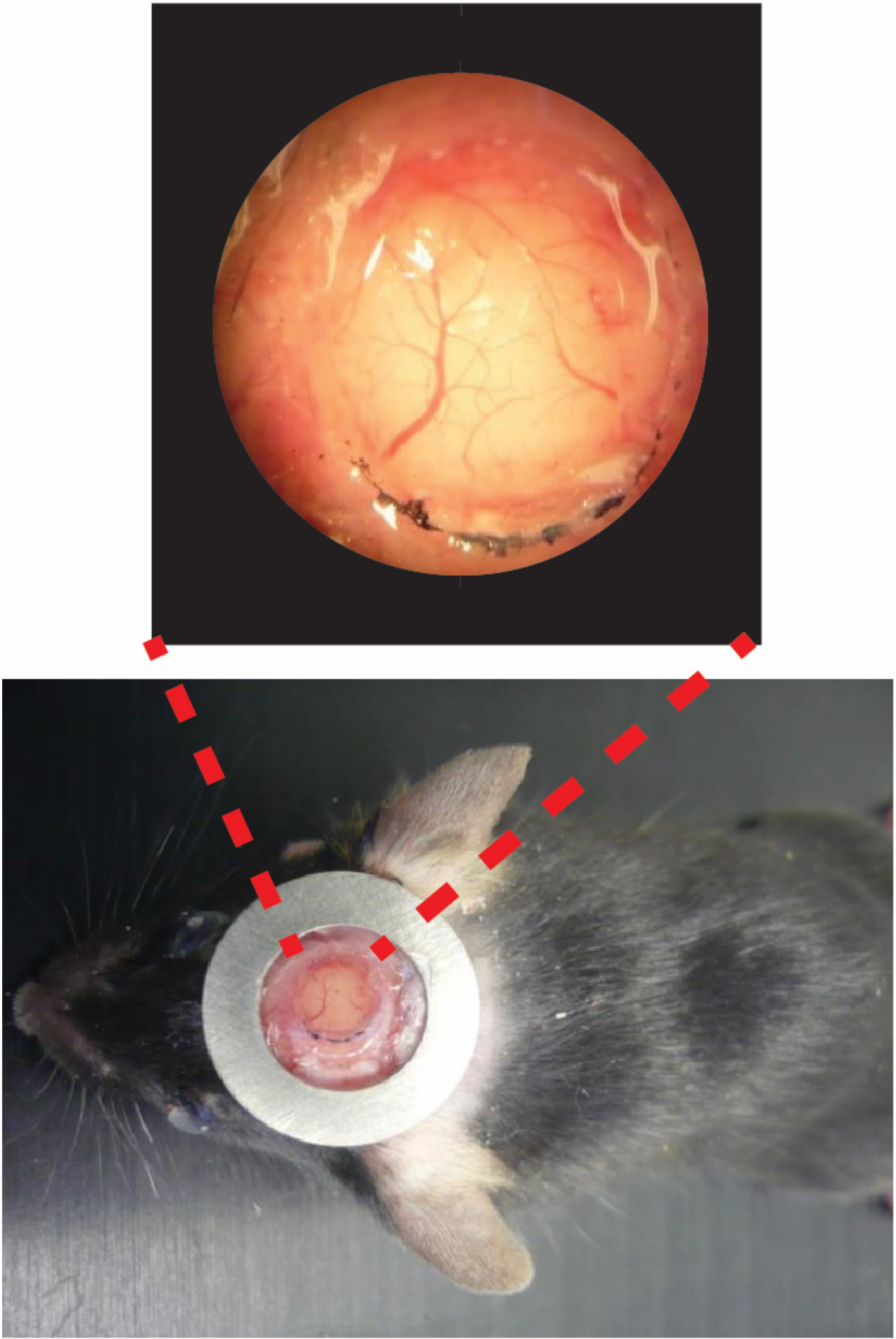

Formula:

COM y~ Vinv:T + Finv:Vinv + (1 | BTIC)

Random effects:

Groups	Variance	Std.Dev.
BTIC	0.003019	0.05495
Residual	0.131593	0.36276

Fixed effects:

	Estimate	Std.	Error t value
(Intercept)	0.38738	0.10306	3.759
Vinv:T*border*	0.84942	0.08389	10.126
Vinv:T*invasive*	0.62006	0.06715	9.233
Vinv:T*diffuse*	0.64303	0.05678	11.324
Vinv:Finv	-1.68903	0.07633	-22.128

Parameter	Definition
COMy	The difference in the centre of mass along the Y axes between its initial value and that at the end of the experiment per unit of time (h) within a position
Vinv	Absolute velocity perpendicular to the tumor border of invading cells within a position
Vret	Absolute velocity perpendicular to the tumor border of retreating cells within a position
Finv	Frequency of invading cells within a position
T	Type of border configuration
BTIC	Type of BTIC cell line
